# m6A-TCPred: a web server to predict tissue-conserved human m^6^A sites using machine learning approach

**DOI:** 10.1186/s12859-024-05738-1

**Published:** 2024-03-25

**Authors:** Gang Tu, Xuan Wang, Rong Xia, Bowen Song

**Affiliations:** 1 Department of Biological Sciences, Xi’an Jiaotong-Liverpool University, Suzhou, 215123 China; 2 Institute of Systems, Molecular and Integrative Biology, University of Liverpool, Liverpool, L7 8TX UK; 3 Department of Financial and Actuarial Mathematics, Xi’an Jiaotong-Liverpool University, Suzhou, 215123 China; 4 Department of Public Health, School of Medicine, Nanjing University of Chinese Medicine, Nanjing, 210023 China

**Keywords:** m^6^A modification, Machine learning, Web server, Support vector machine, Gene ontology

## Abstract

**Background:**

N6-methyladenosine (m^6^A) is the most prevalent post-transcriptional modification in eukaryotic cells that plays a crucial role in regulating various biological processes, and dysregulation of m^6^A status is involved in multiple human diseases including cancer contexts. A number of prediction frameworks have been proposed for high-accuracy identification of putative m^6^A sites, however, none have targeted for direct prediction of tissue-conserved m^6^A modified residues from non-conserved ones at base-resolution level.

**Results:**

We report here m6A-TCPred, a computational tool for predicting tissue-conserved m^6^A residues using m^6^A profiling data from 23 human tissues. By taking advantage of the traditional sequence-based characteristics and additional genome-derived information, m6A-TCPred successfully captured distinct patterns between potentially tissue-conserved m^6^A modifications and non-conserved ones, with an average AUROC of 0.871 and 0.879 tested on cross-validation and independent datasets, respectively.

**Conclusion:**

Our results have been integrated into an online platform: a database holding 268,115 high confidence m^6^A sites with their conserved information across 23 human tissues; and a web server to predict the conserved status of user-provided m^6^A collections. The web interface of m6A-TCPred is freely accessible at: www.rnamd.org/m6ATCPred.

**Supplementary Information:**

The online version contains supplementary material available at 10.1186/s12859-024-05738-1.

## Introduction

In recent years, the field of RNA modification has gained significant prominence, with its origins dating back to the groundbreaking proposal in 2008. To date, over 300 distinct RNA modifications have been identified, with more than 170 of them undergoing extensive investigation [1, 2]. These modifications exert profound influences on RNA molecules, impacting their structural conformation, stability, and functional attributes. Notably, the N6-methyladenosine (m^6^A) modification has garnered substantial attention due to its prevalence within mRNA and its pivotal role in various biological pathways.

m^6^A modification stands out as the most prevalent and comprehensively studied post-transcriptional modification within mRNA, almost across the entire transcriptome. This prevalence is particularly pronounced in higher eukaryotic organisms. The significance of m^6^A modification extends to its far-reaching impact on diverse aspects of biological development, spanning hematopoietic development [3], reproductive processes [4], central nervous system functioning [5] and the regulation of cancer pathways [6]. Consequently, the precise identification of m^6^A methylation sites has become increasingly imperative in the realm of biological research.

Currently, researchers mainly employ two high-throughput sequencing techniques to profile the m^6^A sites: MeRIP-seq and miCLIP-seq. MeRIP-seq capitalizes on m^6^A-specific antibodies to immune-precipitate small RNA fragments following splicing and reverse transcription. Subsequently, the cDNA fragments are sequenced, unveiling the location and extent of m^6^A enrichment [7]. Besides MeRIP-seq, miCLIP-seq utilizes UV light-induced cross-linking to introduce specific mutational features, which enables the precise identification of the m^6^A residues in RNA molecules [8]. Nonetheless, the reliability of both methods has been tested extensively, revealing susceptibility to multiple influencing factors, leading to occasional inaccuracies and instability. These factors include antibody specificity, domain fusion, and various statistical approaches aimed at mitigating technical noise [9–11]. Beyond these technical concerns, the considerable labor, material, and time costs further intensify the challenges faced by researchers.

To address these challenges, computational databases [12–17] and *in silicon* efforts [18–25] focusing on various biological domains have been developed. For m^6^A RNA methylation, tools like *SRAMP* [26] and *iRNA toolkits* [27–29] were established, drawing on a variety of sequence-derived data. The *WHISTLE* [30], emerged as a high-precision predictor, pioneering the integration of domain knowledge and genomic features into m^6^A prediction frameworks. More recently, deep learning-based methodologies have also demonstrated their prowess in m^6^A detection [31–33].While these advancements have significantly enhanced in silico identification of modified residues. To date, no research has been made to predict conserved m^6^A sites across multiple human tissues, despite their established biological significance [34–36]. The ConsRM [37] firstly quantified the conservation degree of base-resolution m^6^A sites between human and mouse transcriptomes. The evolutionary conservation in influenza was researched through potential m^6^A sites based on DRACH motif [38].

The identification of tissue-conserved m^6^A sites assumes paramount importance due to their resistance to interference from extraneous factors and their inherent stability, rendering them invaluable indicators for quantifying m^6^A expression levels. Moreover, most existing prediction tools are predominantly based on sequence information and do not incorporate annotations regarding potential post-transcriptional regulatory functions, which are instrumental in elucidating functional consequences. Therefore, the efficacy of these predictors remains subject to limitations.

**Fig. 1:**
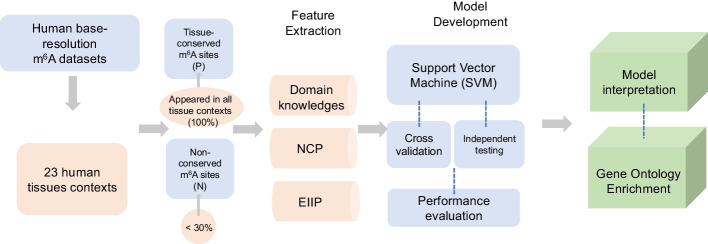
The framework of m6A-TCPred. The entire datasets from 23 human tissue were further classified into conserved and normal datasets. The model constructed on the genomic and sequence features were trained and evaluated through cross validation and independent testing. The model was further illustrated by interpretation and gene ontology enrichment

Here, we present m6A-TCPred, a web server to predict tissue-conserved m^6^A sites in human. By learning and testing the m^6^A datasets identified from 23 human tissues, the newly integrated framework m6A-TCPred (see Fig. 1) provides a high-accuracy mapping of tissue-conserved human m^6^A sites (an average value of AUROC 0.879). Additionally, our results have been integrated into an online platform: a database to hold 268,115 high confidence m^6^A sites with their conserved information across 23 human tissues; and a web server to predict the conserved status of user-uploaded m^6^A datasets. The m6A-TCPred is freely accessible at: www.rnamd.org/m6ATCPred.

## Materials and methods

### Training and testing dataset

m6A-TCPred was proposed to predict the tissue-conserved m^6^A methylation sites. The entire datasets were all high-confidence experimentally validated m^6^A sites (identified in at least two independent studies) collected from m^6^A-Atlas database [39]. To comprehensively expand the prediction range, a total of 268,115 base-resolution m^6^A sites were first filtered from m^6^A-Atlas (with record time > 2). Next, by checking with m^6^A-containing regions identified from 23 different tissue contexts, we defined the m^6^A sites simultaneously appeared in all tissue contexts as tissue-conserved m^6^A sites (dataset P), while the m^6^A sites appeared in less than 30% of tissue contexts as tissue-specific m^6^A sites (dataset N). Importantly, the m^6^A sites that did not appear in any tissue context were excluded to avoid potential bias. Totally, the dataset P contains 10,424 m^6^A sites, while dataset N includes 54,949 m^6^A sites across 23 human tissues. To maximize the use of dataset P, the dataset N was further split into 10 sub-datasets and then developed into 10 models in 1:1 Positive-to-Negative ratio with positive datasets to achieve average performance. For performance evaluation, 80% of the dataset was randomly selected as training data, while the rest of 20% was used for independent testing. Please refer to Additional file 1 for the detailed dataset collected to develop the prediction framework.

### Feature extraction

*Sequence-derived features.* Encoding approaches based on sequence information have been broadly applied and achieved good performance in prediction [40–42]. Our new model also adopted the encoding strategy consisting of two parts: Chemical Properties of nucleotides (NCP) and Electron–Ion Interaction Pseudopotential (EIIP).

The first encoding was originally employed in the prediction of DNA sequence splicing site and its efficiency has been confirmed in RNA modification prediction [43, 44]. It depends on the structural differences of chemical properties from ring structure, functional groups and hydrogen bonds. Specifically, one ring structure exists between cytosine and uracil, as well as two ring structures between adenine and guanine. Adenine and cytosine have amino groups, while ketone group is carried between guanine and uracil. Guanine and cytosine, connected by three hydrogen bonds, have stronger binding ability than adenine and cytosine with two hydrogen bonds. As a result of these three structural chemical properties, the encoding of *i*-th nucleotide of given sequence S will be conducted as Vector S_i_ = (X_i_,Y_i_,Z_i_):

1 $$X_{i} = \left\{ {\begin{array}{*{20}c} 1 & {if\;S_{i} \in \{ A,G\} } \\ 0 & {if\;S_{i} \in \{ C,U\} } \\ \end{array} } \right.,\quad Y_{i} = \left\{ {\begin{array}{*{20}c} 1 & {if\;S_{i} \in \{ A,C\} } \\ 0 & {if\;S_{i} \in \{ G,U\} } \\ \end{array} } \right.,\quad Z_{i} = \left\{ {\begin{array}{*{20}c} 1 & {if\;S_{i} \in \{ A,U\} } \\ 0 & {if\;S_{i} \in \{ C,G\} } \\ \end{array} } \right.$$

Therefore, A, C, G and U can be encoded by vectors (1,1,1), (0,1,0), (1,0,0) and (0,0,1) respectively.

The Electron–Ion Interaction Pseudopotential (EIIP) was calculated from the delocalized electrons energy from amino acids and nucleotides [45]. This encoding strategy was originally used in the DNA sequences to locate exons and gradually promoted to RNA sequences field [46]. In EIIP method, each nucleotide in RNA sequence was standing for a numeric value to represents its EIIP energy. Specifically, the EIIP for nucleotide A, G, C, U is 0.1260, 0.0806, 0.1340 and 0.1335, respectively.

*Genome-derived features.* To capture the distinct characteristics of tissue-conserved m^6^A sites across human tissues, we extracted 54 additional genomic features from m6ALogisticModel package (generation R code in Additional file 5). These features were selected to accurately represent the topological attributes of the modified residues.1–15 are dummy variable features that indicate whether the tissue-conserved m^6^A sites are located within specific transcriptome regions with unique topological properties. All genomic features were generated using the R/Bioconductor package and the hg19 TxDb transcriptome annotation package [47]. In addition, only the primary (longest) transcript of each gene was kept to avoid ambiguities arising from transcript isoforms and extract transcriptional sub-regions [48]. Features 16–19 provide actual valued information on the relative transcript region position, including 3’UTR, 5’UTR, and the whole transcriptome. Sites falling outside these regions are assigned a value of zero. Features 20–27 detail the length of the transcript region containing the modification site, with a value of zero for sites not belonging to the region. Features 28–31 describe the distance between the adenine site and the splicing junction 5’end or 3’end. The distance to the closest tissue-conserved m^6^A site in the training data is calculated to quantify the clustering effect of conserved m^6^A sites. Features 32 and 33 provide the evolutionary conservation score of the conserved adenosine site and its flanking regions, calculated using Phast-Cons score [49] to assess the conservation level of potential nucleotide sequences. The RNA structure surrounding the conserved adenine site is included in features 34–35, predicted using the RNAfold Vienna RNA package [50]. Features 36–43 illustrate the structural function of m^6^A regulatory binding complexes, including readers, writers, and erasers. Features 44–48 encompass genomic properties of genes or transcripts containing conserved m^6^A sites. Features 49–51 indicate the z-score of GC content, while features 52–54 provide omics information, such as microRNA target sites. Detailed information of each genomic feature can be found in Table S1 in Additional file 3.

### Machine learning approach used for prediction of tissue-conserved m^6^A site

The Support Vector Machine (SVM) approach is a data-driven machine learning algorithm widely utilized for classification tasks. Notably, SVM outperforms artificial neural networks in scenarios involving extensive genomic data, yielding lower error rates [51]. This technique has been previously applied to identify biomarkers from gene expression data, explore protein interactions [52], pinpoint therapeutic cancer targets, and achieve genome-wide recognition using diverse high-throughput datasets [53].In this research, the prediction model was constructed on R studio interface of LIBSVM [54], with radial basis function serving as kernel. The other parameters were employed by default.

### Performance evaluation of tissue-conserved m^6^A site prediction

To comprehensively evaluate the performance of our predictor, the SVM classifier was examined through fivefold cross-validation on training datasets and tested from 10 independent testing datasets.

To comprehensively evaluate the prediction model performance, five metrics were introduced as the indicators to examine the reliability. Receiver Operating Characteristic (ROC) curve (sensitivity against 1-specificity) and Area Under ROC Curve (AUROC) are the primary performance evaluation indicators. The other four metrics, Sensitivity (Sn), Specificity (Sp), Matthew’s Correlation Coefficient (MCC) and Overall Accuracy (ACC) were also employed to quantify and test the model reliability. The entire process was conducted in *R language*.

2 $$S_{n} = \frac{TP}{{TP + FN}}$$

3 $$S_{p} = \frac{TN}{{TN + FP}}$$

4 $$MCC = \frac{TP \times TN - FP \times FN}{{\sqrt {(TP + FP) \times (TP + FN) \times (TN + FP) \times (TN + FN)} }}$$

5 $$ACC = \frac{TP + TN}{{TP + TN + FP + FN}}$$

where the TP, TN, FP and FN respectively represent true positive; true negative; false positive and false negative.

### Website construction

The predictor website platform is based on Hyper Text Markup Language (HTML), Cascading Style Sheets (CSS) and Hypertext Preprocessor (PHP), as well as the MySQL tables for metadata storage.

## Results

### Determine the best machine learning sequence strategy and algorithm for tissue-conserved m^6^A site prediction

Although the sequence encoding strategy has already achieved a little reliability in prediction. In order to achieve the best classifier to construct the m6A-TCPred, we combined it with genomic features and tested its performances under different sequence encoding strategies and classifiers on independent dataset (see Table 1). The highest AUROC score is 0.669 when the encoding strategy adopts NCP + EIIP under SVM classifier, representing the best performance among all sequence encoding and algorithm.

**Table 1:** Performance evaluation of tissue-conserved m^6^A sites of using different sequence strategy and algorithms

Sequence Strategy	Algorithm	Independent Testing				
		Sn	Sp	ACC	MCC	AUROC
NCP + ND	SVM	0.598	0.589	0.593	0.187	0.628
	NB	0.655	0.465	0.560	0.123	0.585
	GLM	0.587	0.581	0.584	0.169	0.621
NCP + EIIP	SVM	0.624	0.614	0.619	0.239	0.669
	NB	0.707	0.469	0.585	0.186	0.636
	GLM	0.604	0.619	0.612	0.224	0.660
EIIP + PseKNC	SVM	0.641	0.600	0.620	0.240	0.663
	NB	0.921	0.188	0.555	0.162	0.635
	GLM	0.604	0.606	0.605	0.210	0.648

The SVM (support vector machine) represent binary classification method. NB refers to the naïve bayes classification method. GLM (generalized linear model) is linear regression model. The following sequence encoding strategy, NCP refers to the nucleotide chemical property [43]. ND is nucleotide density [55]. EIIP refers to electron–ion interaction pseudopotential (EIIP) [45]. PseKNC refers to Pseudo K-tuple nucleotide composition [56]

### Performance evaluation of tissue-conserved m^6^A predictor by benchmark and independent testing

All the features are normalized and converted into numerical matrix between 0 and 1. The final tissue-conserved m^6^A prediction model was constructed based on combination of sequence and genomic features.

To comprehensively evaluate the model, ten independent datasets were randomly extracted from negative datasets and integrated with the positive data for average performance evaluation. The prediction model achieved the average value of AUROC 0.879, as well as AUROC 0.871 of fivefold cross-validation (Table 2), suggesting reliability in model distinguishment.

**Table 2:** Prediction performance using cross validation and independent test dataset

Model	Testing method	Sn	Sp	ACC	MCC	AUC
m6A-TCPred	Cross-validation	0.795	0.789	0.792	0.584	0.871
	Independent testing	0.806	0.796	0.801	0.603	0.879

### Feature ranking and functional characterization of tissue-conserved m^6^A sites

**Fig. 2:**
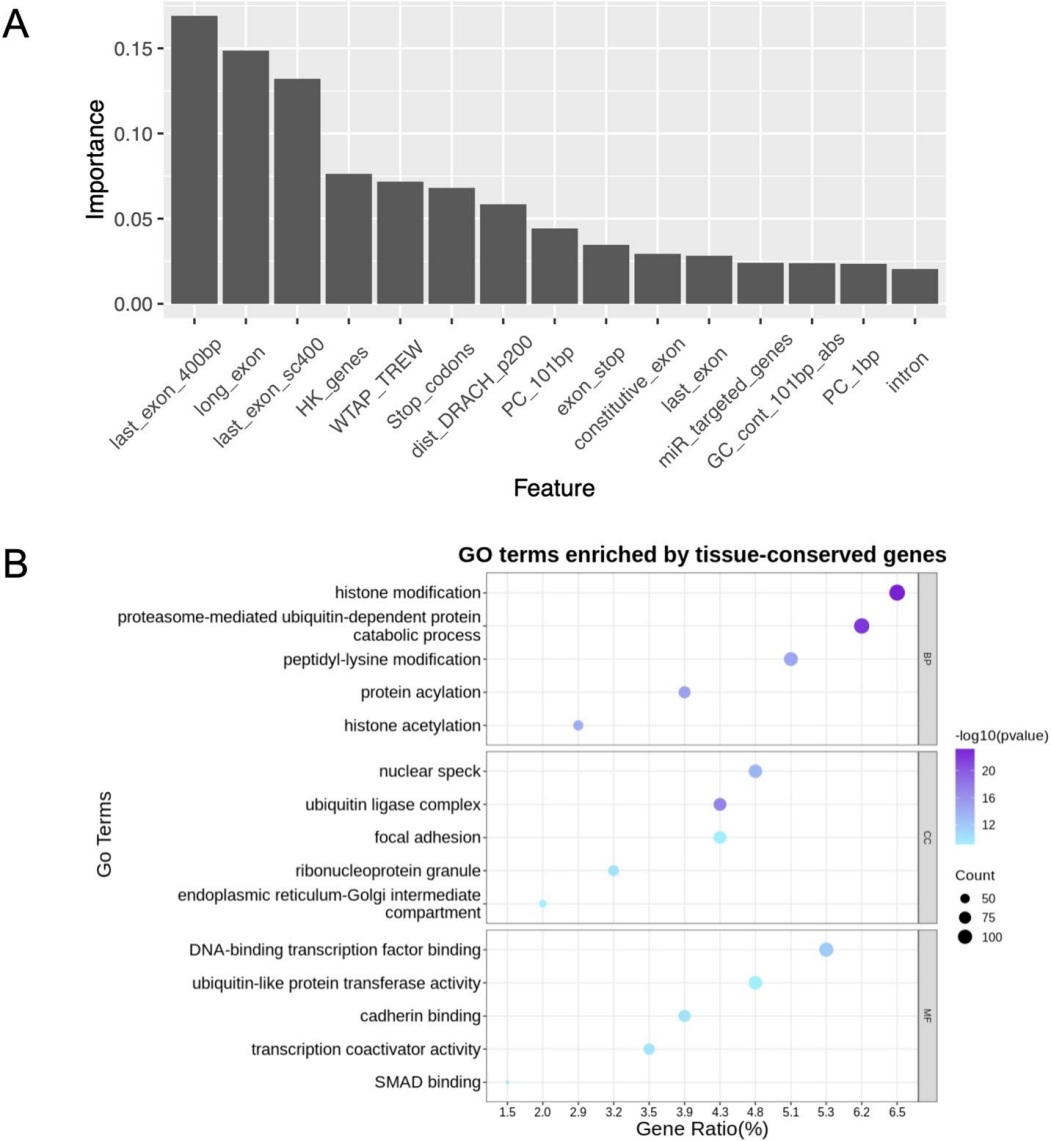
Model interpretation. **A** The top 15 of most contributing features. Features are output based on machine learning model recognition capabilities. **B** Gene enrichment analysis of the tissue-conserved m^6^A sites. BP is the biological process; CC is cell component and MF represents molecular function. The gene identification is obtained by using R package RMAnno. Gene ontology analysis is conducted by R package ClusterProfiler [59]

The feature ranking illustrates the performance efficiency of all features when model processing, which points out the contribution of various features to identifying tissue-conserved m^6^A sites. The Fig. 2A listed the top 15 most effective features in predicting tissue-conserved m^6^A sites, from which we can observed that exon regions may have strong associations in distinguishing tissue-conserved m^6^A sites from non-conserved ones, especially for long exon regions (> 400 bp). Additionally, feature selection was performed. When using the top 24 genomic features, the model exhibited the best performance with an AUROC of 0.89. As more features were added, the model performance slightly decreased, ranging between AUROC of 0.87 and 0.88 (Figure S1 in Additional file 4). Taken together, this result indicated that feature overfitting has only very limited impact on model performance.

We further examined the biological characterization of the tissue-conserved m^6^A sites. Specifically, over 260,000 m^6^A sites were extracted and more than 10,000 of them identified as conserved m^6^A sites in human tissue for examining their putative functional relevance with Gene Ontology (GO) Enrichment Analysis. In Fig. 2B, we present the top five enriched items in biological pathways, cellular composition, and molecular functions, respectively. Many of these enriched items have previously been confirmed to have strong associations with m^6^A methylation. Among them, most of them have been studied in several research. For histone modification, the consumption of H3K36me3 has been proved to decrease the abundance of m^6^A sites [57]. For transcription factor, the m6A-mediated regulation of JUN and JUNB TFs are critical in gene regulation network [58]. A more comprehensive list of the results from the Gene Ontology analysis is available in Additional file 2 (Fig. 2).

Additionally, to further explore the molecular features of tissue-conserved m^6^A sites from normal ones, we conducted the motif analysis for both of them. The result (Figure S2 in Additional file 4) showed that the sequences of tissue-conserved m^6^A sites and normal m^6^A sites follow the pattern of DRACH motif. Meanwhile, no significant differences were observed between tissue-conserved and non-conserved m^6^A sites, which was consistent with our finding that sequence-based information alone cannot effectively used for classification.

### Web server implementation

**Fig. 3:**
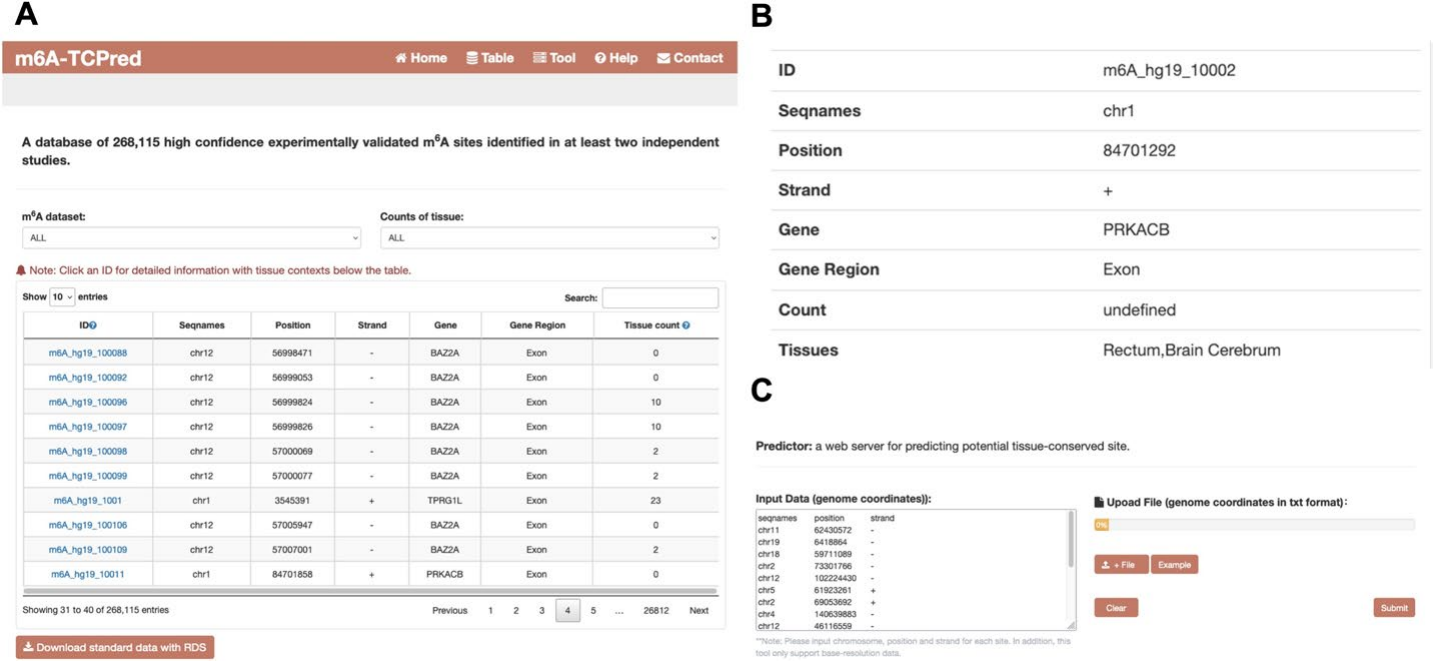
The database and web server of m6A-TCPred. **A** The database exhibited the information of all m^6^A sites. The user can select conserved, non-conserved sites and tissue counts or search according to their own needs. **B** The detailed information of one m^6^A site. **C** The web server allows users to submit their own m^6^A coordinates. The m6A-TCPred will predict conservative probability of each site

To enhance the practicality and accessibility of our prediction model, we have developed a user-friendly web server, which is accessible at http://www.rnamd.org/m6ATCPred. Figure 3A exhibits all m^6^A site datasets incorporated in our model. The selection menus provide users with the flexibility to filter conserved, non-conserved sites, or examine the tissue counts for each site. Detailed information for each site is easily accessible by clicking on the respective ID (see Fig. 3B). Figure 3C illustrates the functionality of our web server, it allows users to upload their m^6^A coordinates, while the possibility of input data is evaluated, and each related site is classified into conserved/non-conserved. The free download function is also available.

## Conclusion

m^6^A methylation stands as one of the most pivotal RNA modifications, with its substantial abundance in mRNA and integral role in biological processes garnering significant attention. Despite this, the precise identification and localization of conserved m^6^A sites in human tissues has remained a largely unexplored domain, primarily due to limitations associated with experimental methodologies. In response to these challenges, our research has developed m6A-TCPred, a computational predictor that integrates sequence feature information and genomic features. In contrast to existing predictors, our model excels in the accurate identification of tissue-conserved m^6^A sites and their discrimination from non-conserved sites across multiple human tissues. Meanwhile, regrading whether tissue-conserved sites are influenced by housekeeping gene, we evaluated the model performance by adjusting datasets that does not contain housekeeping gene. Our result (Table S2 in Additional file 3) showed there is no significant difference compared with original AUROC. Therefore, the m6A-TCPred didn’t have any bias on housekeeping gene.

Our research findings are exciting and the achievements are mainly concentrated on three aspects. Firstly, the m6A-TCPred is a high-accuracy predictor, demonstrating the high efficiency in predicting tissue-conserved m^6^A sites. Through fivefold cross-validation and independent testing, our model achieves an impressive AUROC score of 0.879, surpassing the previous limitations associated with sequence encoding. To ensure the broad accessibility of our research, we have integrated the entire model into a user-friendly website. This resource is open to all, enabling individuals to submit genome coordinate files and use our predictor for tissue-conserved m^6^A site predictions. We anticipate that this tool will serve as a powerful resource for researchers delving into the intricacies of conserved m^6^A sites in human tissue. Secondly, the model ranking and GO analysis provides biological characterizations of tissue-conserved m^6^A sites. It identified the potential functional regions with high probability of conserved m^6^A sites. The GO analysis provides a connection between conserved m^6^A sites and biological functions and some of the content has been mentioned in relevant research. Thirdly, the whole dataset and its relevant annotations were integrated into a website, which is the first collection about tissue-conserved m^6^A sites.

It is essential to acknowledge certain limitations. The presence of bias in our training datasets, stemming from inherent limitations in experimental techniques, may influence the model's performance. Further, sample size constraints might not capture the full spectrum of potential outcomes. As we continue our research, we remain committed to improving the model's reliability with the incorporation of the latest sequencing data. Additionally, we recognize that the accuracy of our predictions is contingent on the availability of additional information, such as RNA secondary structure, free energy, RNA type, and more.

### Supplementary Information

**Additional file 1. Supplementary Table S1.** m6A sites distributions in different tissues

**Additional file 2. Supplementary Table S2.** The detailed information of Gene Ontology analysis

**Additional file 3. Table S1.** Genome-derived features from m6A datasets. **Table S2.** The model performance of non-HKG m6A sites

**Additional file 4. Figure S1.** Feature selection of genome-derived features of m6ATCPred. **Figure S2**. The motif analysis of conserved m6A sites. A) The motif of tissue-conserved m6A residues. B) The motif of normal m6A restudies.

**Additional file 5**. #Genomic Feature genertaion.

## Data Availability

The raw data used to construct m6A-TCPred was collected from the m6A-Atlas v2.0: http://rnamd.org/m6a. The detailed information of m6A-TCPred dataset can be found in Supplementary information Additional file 1 and is freely accessible at: http://www.rnamd.org/m6ATCPred.

## Abbreviations

m^6^A: N6-methyladenosine
NCP: Nucleotide chemical property
EIIP: Electron–ion interaction pseudopotential
SVM: Support vector machine
Sn: Sensitivity
Sp: Specificity
MCC: Matthews correlation coefficient
ACC: Overall accuracy
AUROC: Area under the ROC curve
GO: Gene ontology
BP: Biological process
CC: Cell component
MF: Molecular function
HTML: Hyper text markup language
CSS: Cascading style sheets
PHP: Hypertext preprocessor
