# Supplementary material for: m6A-TCPred: a web server to predict tissue-conserved human m6A sites using machine learning approach
Source: BMC Bioinformatics. 2024 Mar 25;25:127. doi: 10.1186/s12859-024-05738-1 (PMC10962094; doi:10.1186/s12859-024-05738-1)
Supplement: Supplementary file 3 — Additional file 3. Table S1. Genome-derived features from m6A datasets. Table S2. The model performance of non-HKG m6A sites [file 12859_2024_5738_MOESM3_ESM.docx]

**Supplementary Table**

**Table S1 Genome-derived features from m6A datasets**

| ID | Name | Description | Note |
| --- | --- | --- | --- |
| 1 | UTR5 | 5' UTR | Dummy variables indicating whether the site is overlapped to the topological region on the major RNA transcript. |
| 2 | UTR3 | 3' UTR |  |
| 3 | cds | Coding sequence |  |
| 4 | Start_codons | start codons flanked by 100bp |  |
| 5 | Stop_codons | Stop codons flanked by 100bp |  |
| 6 | TSS_A | downstream 100bp of TSS on A |  |
| 7 | exon_stop | exons containing stop codons |  |
| 8 | alternative_exon | alternative exons |  |
| 9 | constitutive_exon | constitutive exons |  |
| 10 | internal_exon | Internal exons |  |
| 11 | long_exon | long exons (exon length >= 400bp) |  |
| 12 | last_exon | 5’ last_exon |  |
| 13 | last_exon_400bp | 5’ 400bp of the last exons |  |
| 14 | last_exon_sc400 | 5’ 400bp of the last exons containing stop codons |  |
| 15 | intron | intron |  |
| 16 | pos_cds | relative position on coding sequence | Relative position on the region |
| 17 | pos_UTR5 | relative position on 5'UTR |  |
| 18 | pos_UTR3 | relative position on 3'UTR |  |
| 19 | pos_exons | relative position on exon |  |
| 20 | length_UTR5 | 5'UTR length | The region length in bp. |
| 21 | length_UTR3 | 3'UTR length |  |
| 22 | length_gene_ex | mature transcript length |  |
| 23 | length_cds | Coding sequence length |  |
| 24 | length_gene_full | full transcript length |  |
| 25 | Length_tx_exon | transcript exonic length |  |
| 26 | Length_tx_full | transcript full length |  |
| 27 | DRACH | motif | motif |
| 28 | dist_sj_5_p2000 | distance to the 5' splicing junction | Nucleotide distances toward the splicing junctions or the nearest neighboring sites. |
| 29 | dist_sj_3_p2000 | distance to the 3' splicing junction |  |
| 30 | Dist_DRACH_p2000 | distance to the nearest DRACH motif (peaked at 2000bp) | Distance to DRACH motif |
| 31 | Dist_DRACH_p200 | distance to the nearest DRACH motif (peaked at 200bp) |  |
| 32 | PC_1bp | phastCons scores of the nucleotide | Scores related to evolutionary conservation |
| 33 | PC_101bp | average phastCons scores within the flanking 50bp |  |
| 34 | struc_hybridize | Predicted RNA hybridized region | RNA secondary structures |
| 35 | struc_loop | Predicted RNA loop region |  |
| 36 | YTHDC1_TREW | YTH family | m^6^A regulatory binding features |
| 37 | YTHDF1_TREW |  |  |
| 38 | YTHDC2_TREW |  |  |
| 39 | WTAP_TREW | methyltransferase complex |  |
| 40 | METTL16_CLIP |  |  |
| 41 | ALKBH5_PARCLIP | demethylases complex |  |
| 42 | FTO_CLIP |  |  |
| 43 | FTO_eCLIP |  |  |
| 44 | sncRNA | sncRNA | Genomic properties |
| 45 | lncRNA | lncRNA |  |
| 46 | HK_genes | housekeeping genes |  |
| 47 | isoform_num | Number of isoform |  |
| 48 | exon_num | Number of exon |  |
| 49 | GC_cont_genes | gene level GC content z score | GC content related feature |
| 50 | GC_cont_101bp | 101bp GC content z score |  |
| 51 | GC_cont_101bp_abs | absolute value of the 101bp GC content z score |  |
| 52 | Verified_miRtargets | miRNA targeted sites verified by experiment | Attributes of the genes or transcripts |
| 53 | TargetScan | Predicted miRNA targeted sites by TargetScan |  |
| 54 | miR_targeted_genes | miRNA targeted genes |  |

Table S2. The model performance of non-HKG m6A sites

| Model | Testing method | Sn | Sp | ACC | MCC | AUC |
| --- | --- | --- | --- | --- | --- | --- |
| non-HKG | Independent testing | 0.797 | 0.806 | 0.801 | 0.603 | 0.874 |

Note: Sensitivity (Sn), Specificity (Sp), Matthew’s Correlation Coefficient (MCC) and Overall Accuracy (ACC).14,106 m6A sites on were HKG deleted, of which 3,778 were from the positive dataset and 10,382 were from negative dataset. As a result of this, remaining 6646 m6A sites are in positive dataset and 44618 are m6A sites in negative dataset. The model extracts 80% m6A sites from the new positive dataset to form a training dataset with 1:1 ratio from the negative dataset. The remaining data were used for testing. The model performance takes the average value of AUROC of 10 times training.
