# Supplementary material for: m6A-TCPred: a web server to predict tissue-conserved human m6A sites using machine learning approach
Source: BMC Bioinformatics. 2024 Mar 25;25:127. doi: 10.1186/s12859-024-05738-1 (PMC10962094; doi:10.1186/s12859-024-05738-1)
Supplement: Supplementary file 4 — Additional file 4. Figure S1. Feature selection of genome-derived features of m6ATCPred. Figure S2. The motif analysis of conserved m6A sites. A) The motif of tissue-conserved m6A residues. B) The motif of normal m6A restudies. [file 12859_2024_5738_MOESM4_ESM.docx]

**Supplementary Figures**
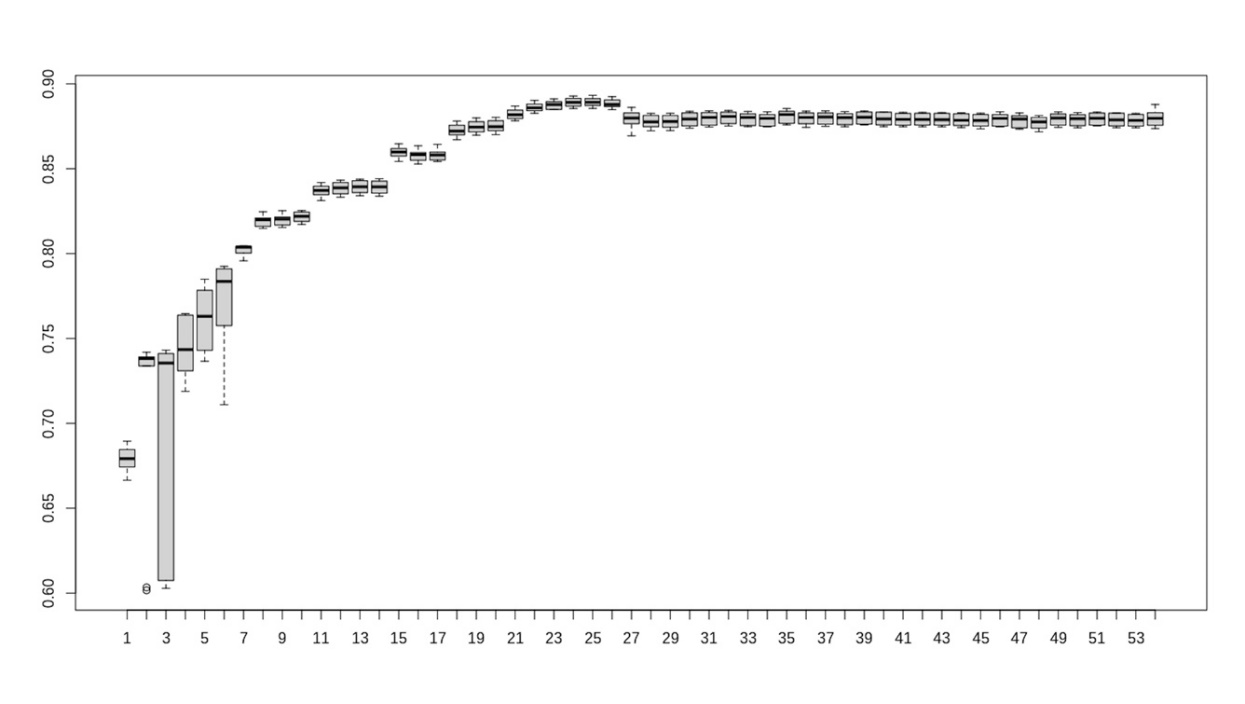


Figure S1. Feature selection of genome-derived features of m6ATCPred.


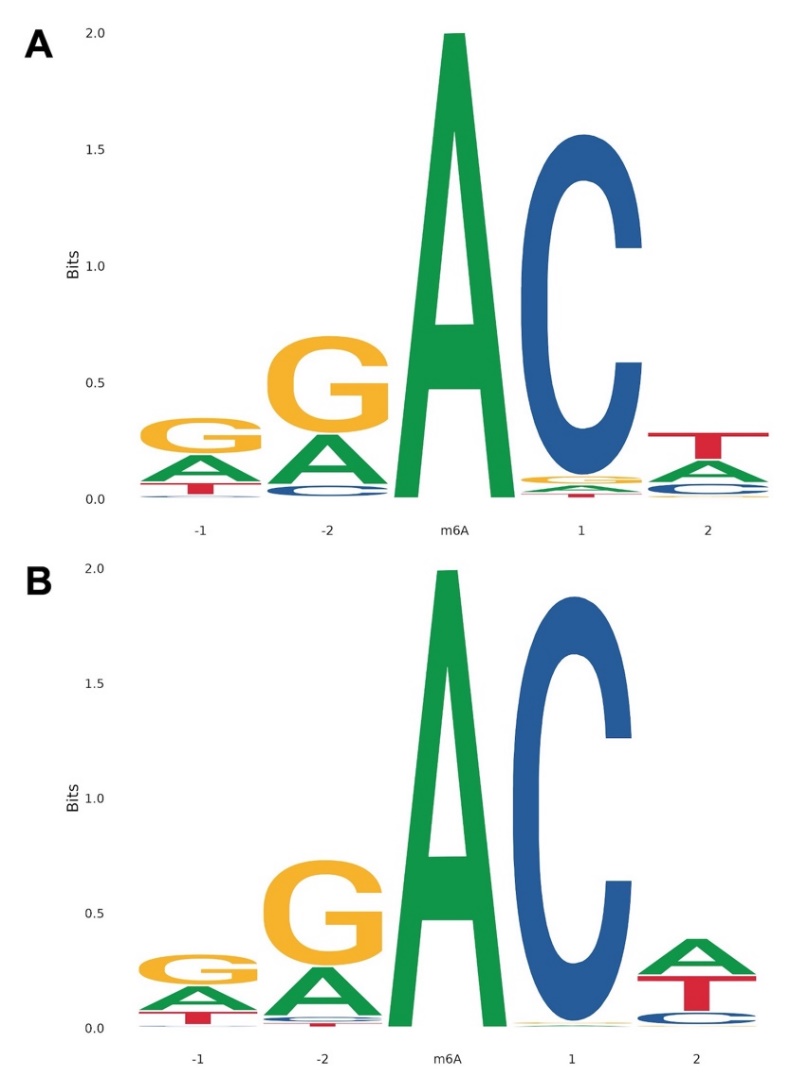


Figure S2. The motif analysis of conserved m6A sites. A) The motif of tissue-conserved m6A residues. B) The motif of normal m6A restudies.
